# Disease flares in rheumatoid arthritis are associated with joint damage progression and disability: 10-year results from the BeSt study

**DOI:** 10.1186/s13075-015-0730-2

**Published:** 2015-08-31

**Authors:** Iris M. Markusse, Linda Dirven, Andreas H. Gerards, Johannes H L M van Groenendael, H. Karel Ronday, Pit J S M Kerstens, Willem F. Lems, Tom W J Huizinga, Cornelia F. Allaart

**Affiliations:** Department of Rheumatology, Leiden University Medical Center, Leiden, The Netherlands; Department of Rheumatology, Vlietland Hospital, Schiedam, The Netherlands; Department of Rheumatology, Fransiscus Hospital, Roosendaal, The Netherlands; Department of Rheumatology, Haga Hospital, the Hague, The Netherlands; Department of Rheumatology, Reade, Amsterdam, The Netherlands; Department of Rheumatology, VU Medical Center, Amsterdam, The Netherlands

## Abstract

**Introduction:**

Flares in patients with rheumatoid arthritis are suggested to sometimes spontaneously resolve. Targeted therapy could then entail possible overtreatment. We aimed to determine the flare prevalence in patients who are treated-to-target and to evaluate associations between flares and patient-reported outcomes and radiographic progression.

**Methods:**

In the BeSt study, 508 patients were treated-to-target for 10 years. After initial treatment adjustments to achieve disease activity score ≤2.4, a flare was defined from the second year of follow-up onwards, according to three definitions. The first definition is a disease activity score >2.4 with an increase of ≥0.6 regardless of the previous disease activity score. The other definitions will be described in the manuscript.

**Results:**

The flare prevalence was 4–11 % per visit; 67 % of the patients experienced ≥1 flare during 9 years of treatment (median 0 per patient per year). During a flare, functional ability decreased with a mean difference of 0.25 in health assessment questionnaire (*p* < 0.001), and the odds ratios (95 % confidence intervals) for an increase in patients’ assessment of disease activity, pain and morning stiffness of ≥20 mm on a visual analogue scale were 8.5 (7.3–9.8), 8.4 (7.2–9.7) and 5.6 (4.8–6.6), respectively, compared to the absence of a flare. The odds ratio for radiographic progression was 1.7 (1.1–2.8) in a year with a flare compared to a year without a flare. The more flares a patient experienced, the higher the health assessment questionnaire at year 10 (*p* < 0.001) and the more radiographic progression from baseline to year 10 (*p* = 0.005).

**Conclusion:**

Flares were associated with concurrent increase in patient’s assessment of disease activity, pain and morning stiffness, functional deterioration and development of radiographic progression with a dose–response-effect, both during the flare and long term. This suggests that intensifying treatment during a flare outweighs the risk of possible overtreatment.

**Trial registration:**

Dutch trial registry NTR262 (7 September 2005) and NTR265 (8 September 2005).

## Introduction

Despite current effective treatment of patients with rheumatoid arthritis (RA), episodes of increased disease activity still may occur [1, 2]. These episodes are generally referred to as ‘flares’. What constitutes a flare can be a matter for debate. This partly hinges on the fact that patients may experience a flare which, for logistic or other reasons, cannot be registered as an increase in disease activity. Therefore the notion of a flare is challenging [1–3]. It is generally understood, though, that flares are associated with concurrent deterioration of patient-reported outcomes. When the increase in disease activity is registered, flares are often managed with treatment intensification [3–6]. Following a treat-to-target strategy, treatment should be adjusted when a predefined target has not been achieved or maintained. It has proven to be effective in trials [7, 8], and is under the concept of ‘tight control’ also adopted in the recommendations for daily practice [9, 10]. However, flares may also spontaneously resolve. In that case, targeted therapy entails the possibility of overtreatment.

In the BeSt study, treatment was targeted at low disease activity (disease activity score (DAS) ≤2.4, using the original DAS based on a 53/44 joints assessment), with 3-monthly DAS measurements over 10 years. In this post-hoc analysis, we aimed to examine the prevalence of flares defined by increases in disease activity, and to determine the short-term and long-term effects of these flares on radiographic progression, function and patient-reported outcomes.

## Methods

### Patients

The multicenter, clinical trial BeSt (Dutch acronym for treatment strategies) with 10-year follow-up enrolled 508 patients with recent-onset, active RA according to the 1987 criteria [11]. Treatment adjustments were made based on 3-monthly DAS measurements, targeted at low disease activity (DAS ≤2.4). If DAS was >2.4, medication was intensified. As long as the DAS was ≤2.4 (from at least 6 months), combination therapy was tapered to monotherapy (usually methotrexate monotherapy), and then monotherapy was tapered to a maintenance dose. When DAS was <1.6 for at least 6 months during a maintenance dose, medication was discontinued, but as soon as DAS increased to >1.6, the last effective medication was restarted and, when DAS increased to >2.4, treatment was further intensified. The study protocol was approved by the medical ethics committees of all participating centers (listed in the Acknowledgements) and all patients gave written informed consent. More details on the BeSt study protocol were previously published [12].

### End points

During year 1, initial treatment adjustments were made to achieve the target of low disease activity in most patients. From the second year on, the presence or absence of a flare was defined per visit. No unambiguous definition of a flare is yet established but, recently, a flare definition based on the 28-joint DAS (DAS28) was validated [5]. From this definition, we derived three definitions of flare based on the 44-joint DAS. Our definitions are partially overlapping, but were always tested separately. ‘Flare A’ was defined as DAS >2.4, with an increase in DAS of at least 0.6 from a previous DAS of any value. A ‘minor flare B’ was defined as DAS >2.4, from a previous DAS ≤2.4 with an increase in DAS <0.6, and a ‘major flare B’ as DAS >2.4 from a previous DAS ≤2.4 with an increase in DAS of ≥0.6.

The cut-off for DAS of 2.4 was chosen based on the target of the BeSt study. The cut-off for the difference in DAS of 0.6 was based on the European League Against Rheumatism (EULAR) criteria, where a decrease of >0.6 in DAS is stated as a (clinically relevant) response [13]. Consequently, we classified a ≥0.6 increase in DAS as a (clinically relevant) deterioration. It is unknown whether we should take into account the absolute value of the previous DAS (thus, not only the change in DAS) when defining a flare. Therefore, flare A and major flare B were distinguished.

Functional ability was measured 3-monthly using the health assessment questionnaire (HAQ; range 0–3) [14]. An improvement of 0.22 in HAQ is considered to represent a clinically relevant improvement [15]. Hence, an increase of 0.22 was considered to be a clinically relevant deterioration. At every visit, patients filled in several visual analogue scales (VAS; range 0–100 mm), assessing general health (VASgh), disease activity (VASda), pain (VASpain) and morning stiffness (VASms). As VASgh is part of the DAS calculation [16], this score was not used for further analysis. A clinically relevant cut-off for an increase in VAS of at least 20 mm difference, as proposed by Khan et al. [17], was used to test whether patients during a flare report higher VAS scores than in situations without a flare.

Joint damage progression was assessed on radiographs of hands and feet using the Sharp/ van der Heijde score (SHS; range 0–448) [18]. Radiographs were obtained yearly and were scored in one session, in random order, by two blinded readers. Radiographic progression was defined as an increase in SHS of >0.5 during a year.

During the trial, treatment adjustments were registered in a separate ‘monitoring database’. Due to different formats the ‘general’ and ‘monitoring databases’ cannot be readily connected for analysis. Therefore, three samples of 100 patients who experienced a flare A, minor flare B and major flare B, were randomly selected and, for these patients, data from both databases were manually combined to explore the relationship between occurrences of flares and previous and subsequent treatment adjustments.

### Statistical analysis

Descriptive statistics were used to determine the frequency of flares.

All analyses were performed separately for the several definitions of flare. Patients with flare A were compared to patients without flare A; patients with major flare B were compared to patients with minor flare B and patients with no flare B. Associations between flares and functional ability, joint damage and VAS were tested using mixed models, which is a robust method since it takes into account all patients and can also handle missing data not completely at random. HAQ was compared over time between patients with and without a flare per visit, with a linear mixed model (LMM). Flare, time and its interaction term were entered as determinants. A Toeplitz covariance matrix was used, because this best fitted the data based on the log likelihood ratio test.

To evaluate long-term and dose–response effects of the occurrence of a flare, patients were categorized according to the number of flares experienced during follow-up (none, 1, 2, or ≥3 flares for each definition of flare). Mean HAQ during follow-up was compared between the categories, as well as the HAQ at year 10 (based on a completers analysis). A Kruskal-Wallis test was performed because of a non-Gaussian distribution of the outcome variables. Cumulative probability plots for mean HAQ during years 2 to 10 and radiographic progression over 10 years were created to visualize the differences between these categories.

For each VAS type, the difference between two subsequent scores was calculated. Percentages of patients with an increase of ≥20 mm between two subsequent visits were reported [17]. A generalized linear mixed model (GLMM) was used to calculate the odds ratio for an increase of ≥20 mm in VAS score (from the preceding visit) during a flare. Flare and time were entered as determinants. Separate GLMM were performed for an increase of ≥20 mm (yes/no) in VASda, VASpain and VASms as outcomes. Covariance matrices were chosen based on their fit to the data, resulting in the Toeplitz covariance matrix for VASda and the Identity covariance matrix for VASpain and VASms.

SHS progression was evaluated yearly. In every patient, for every year, the occurrence of a flare was checked. We tested whether the occurrence of a flare was associated with radiographic progression (defined as an increase in SHS >0.5 during that year, yes/no). A GLMM with an Identity covariance matrix was used. Flare, time and its interaction term were again entered as determinants. To compare SHS progression over 10 years in patients who ever had a flare with those who never experienced a flare, a Kruskal-Wallis test was used (based on a completers analysis). Also, radiographic progression was compared between the categories based on the numbers of flares per patient (none, 1, 2, ≥3 flares).

## Results

### Frequency of flares

In 480 patients, sufficient follow-up data were available to define presence or absence of a flare during at least one visit. At baseline, patients had active disease with a mean (standard deviation (SD)) DAS of 4.4 (0.9) and a mean HAQ of 1.4 (0.7). During the first year of follow-up, disease activity was increasingly suppressed. At the first visit during year 2, patients had a mean DAS of 2.0 (1.0), a HAQ of 0.6 (0.6), and 320/480 patients (67 %) had achieved a DAS ≤2.4.

**Fig. 1:**
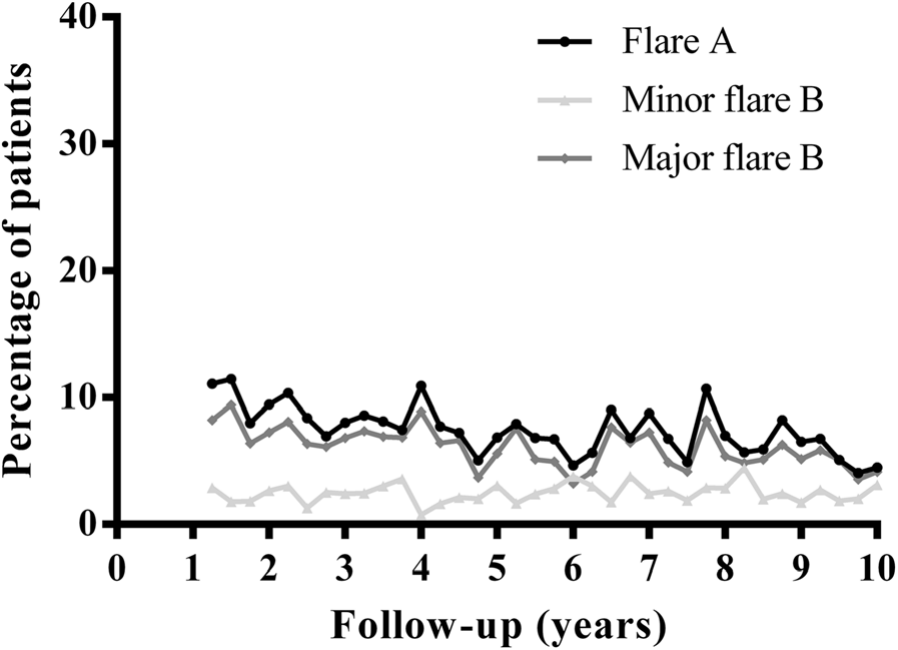
Percentage of patients with a flare per performed visit over time. Flare A: from any DAS to DAS >2.4 with an increase in DAS of ≥0.6; minor flare B: from DAS ≤2.4 to DAS >2.4 with an increase in DAS of <0.6; major flare B: from DAS ≤2.4 to DAS >2.4 with an increase in DAS of ≥0.6. Note, flares are defined from year 2 to year 10

During years 2 to 10, the majority of patients experienced one or more flares. The prevalence of flares in accomplished study visits decreased over time, for all three definitions (Fig. 1). We found a prevalence of flare A of 4–11 % per visit over time. Comparable frequencies were found for major flare B (prevalence 4–9 %) and a minor flare B occurred less often (prevalence 1–6 %). Flare A occurred at least once in 321/480 patients (67 %). A minor flare B occurred in 159/480 patients (33 %) and a major flare B in 304/480 patients (63 %). In patients who experienced at least one flare A, the median (interquartile range (IQR)) number of flares during follow-up was 2 (1–4). For a minor flare B, this was 0 (0–1) and for major flare B this was 2 (1–3).

When evaluating the percentage of patients who ever achieved remission or low disease activity during total follow-up, comparable percentages were found in patients ever having a flare and patients never having a flare (regardless of definition of flare, data not shown).

**Fig. 2:**
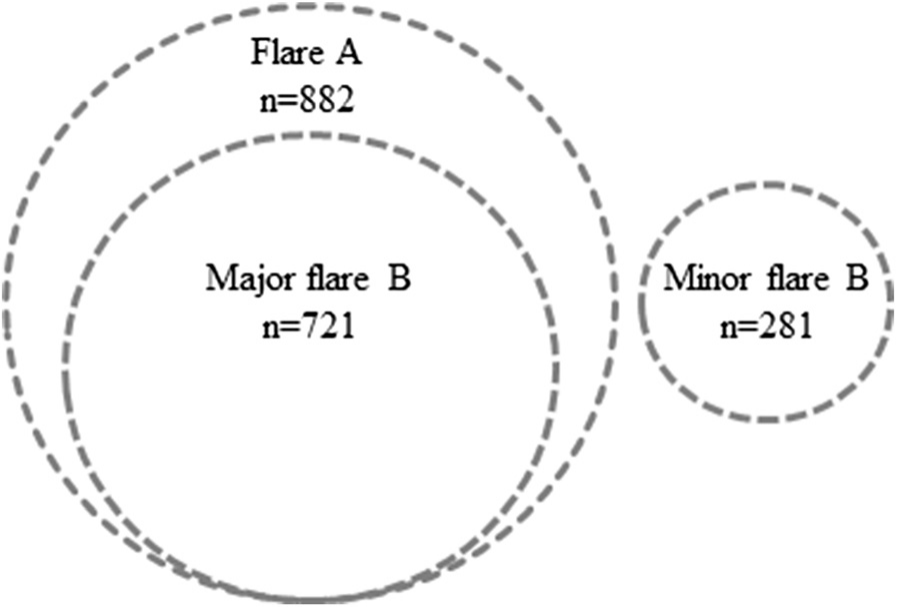
Total number of flares during year 2 to year 10 in all patients (n = 480), according to the following definitions: Flare A (n = 882/11,458): from any DAS to DAS >2.4 with an increase in DAS ≥0.6; Minor flare B (n = 281/11,458): from DAS ≤2.4 to DAS >2.4 with an increase in DAS <0.6; Major flare B (n = 721/11,458): from DAS ≤2.4 to DAS >2.4 with an increase in DAS ≥0.6. It indicates the concordance and discordance between the definitions of flare. Note, more than one flare according to the same definition or according to another definition can occur in the same patient

Table 1 shows the number of patients that experienced none, 1, 2 and ≥3 flares during follow-up according to all definitions. The circles in Fig. 2 represent the total of visits in which presence or absence of flares was defined, indicating the concordance and discordance between the definitions.

**Table 1:** Dose–response effect of the number of flares on functional ability and radiographic progression

	Frequency,	HAQ	HAQ	SHS progression baseline to year 10
	n (%) patients	year 2 to year 10	at year 10	
	Total n = 480	median (IQR)	median (IQR)	median (IQR)
No. of flare A				
0	159 (33)	0.2 (0.0–0.6)	0.0 (0.0–0.5)	1.3 (0.0–3.1)
1	100 (21)	0.5 (0.2–0.9)	0.4 (0.0–0.9)	2.3 (0.5–9.6)
2	73 (15)	0.6 (0.3–0.9)	0.6 (0.1–0.9)	3.0 (0.0–10.0)
≥3	148 (31)	0.8 (0.4–1.1)	0.8 (0.4–1.3)	4.3 (0.5–20.1)
* p* value		<0.001	<0.001	0.005
No. of minor flare B				
0	321 (67)	0.4 (0.1–0.8)	0.3 (0.0–0.9)	2.0 (0.0–6.9)
1	89 (19)	0.6 (0.4–1.0)	0.6 (0.3–1.0)	6.0 (0.5–26.0)
2	37 (8)	0.7 (0.3–1.1)	0.8 (0.3–1.1)	4.5 (0.1–26.8)
≥3	33 (7)	0.9 (0.6–1.1)	0.8 (0.4–1.3)	1.0 (0.0–13.5)
* p* value		<0.001	<0.001	0.026
No. of major flare B				
0	176 (37)	0.3 (0.0–0.8)	0.0 (0.0–0.8)	1.5 (0.0–3.5)
1	114 (24)	0.5 (0.2–0.9)	0.4 (0.0–0.9)	2.0 (0.5–10.3)
2	68 (14)	0.6 (0.3–0.9)	0.6 (0.1–0.9)	3.5 (0.5–17.0)
≥3	122 (25)	0.8 (0.4–1.1)	0.8 (0.3–1.1)	4.5 (0.3–17.3)
* p* value		<0.001	<0.001	0.009

Due to drop out, not all patients had a HAQ at year 10 available, or a SHS progression score from baseline to year 10

‘Flare A’ defined as DAS >2.4, with an increase in DAS of at least 0.6 from a previous DAS of any value. ‘Minor flare B’ defined as DAS >2.4, from a previous DAS ≤2.4 with an increase of DAS <0.6. ‘Major flare B’ defined as DAS >2.4 from a previous DAS ≤2.4 with an increase in DAS ≥0.6

*IQR* interquartile range, *HAQ* health assessment questionnaire, *SHS* Sharp/ van der Heijde score

### Treatment

Exploration of three samples of 100 randomly selected patients with flare A, minor flare B and major B demonstrated that only approximately 25 % of flares were preceded by medication tapering. Although the study protocol dictated to change medication or to increase the dose in case of DAS >2.4, this was done only in ±60 % of flares. In particular, in 11 % of cases of a minor flare B, rheumatologists scheduled an extra visit 1 month later, hoping to find that the flare had resolved spontaneously. This indeed occurred in 73 % of those situations. At the next evaluation following a flare, a DAS ≤2.4 was achieved again in 60 % of patients. According to our definition of flare, the remaining 40 % of patients could have a flare A at this evaluation, not a flare B (as this definition required a previous DAS of ≤2.4).

### Functional ability

The mean (SD) HAQ in patients at a visit with a flare A was 1.04 (0.63), and in patients at a visit with no flare A 0.53 (0.56). Patients with a minor flare B and a major flare B had a mean HAQ of 0.85 (0.55) and 0.96 (0.60), respectively, compared to 0.53 (0.57) for patients with no flare B. Following the LMM, compared to the absence of a flare, a flare A was associated with an increase in HAQ of 0.251 (*p* < 0.001). Compared to the absence of a flare, a minor flare B was associated with an increase in HAQ of 0.059 (*p* = 0.001), while a major flare B was accompanied by an increase in HAQ of 0.226 (*p* < 0.001). The difference in functional ability at the time of a minor flare compared to a major flare was small (mean difference in HAQ 0.167; *p* < 0.001).

**Fig. 3:**
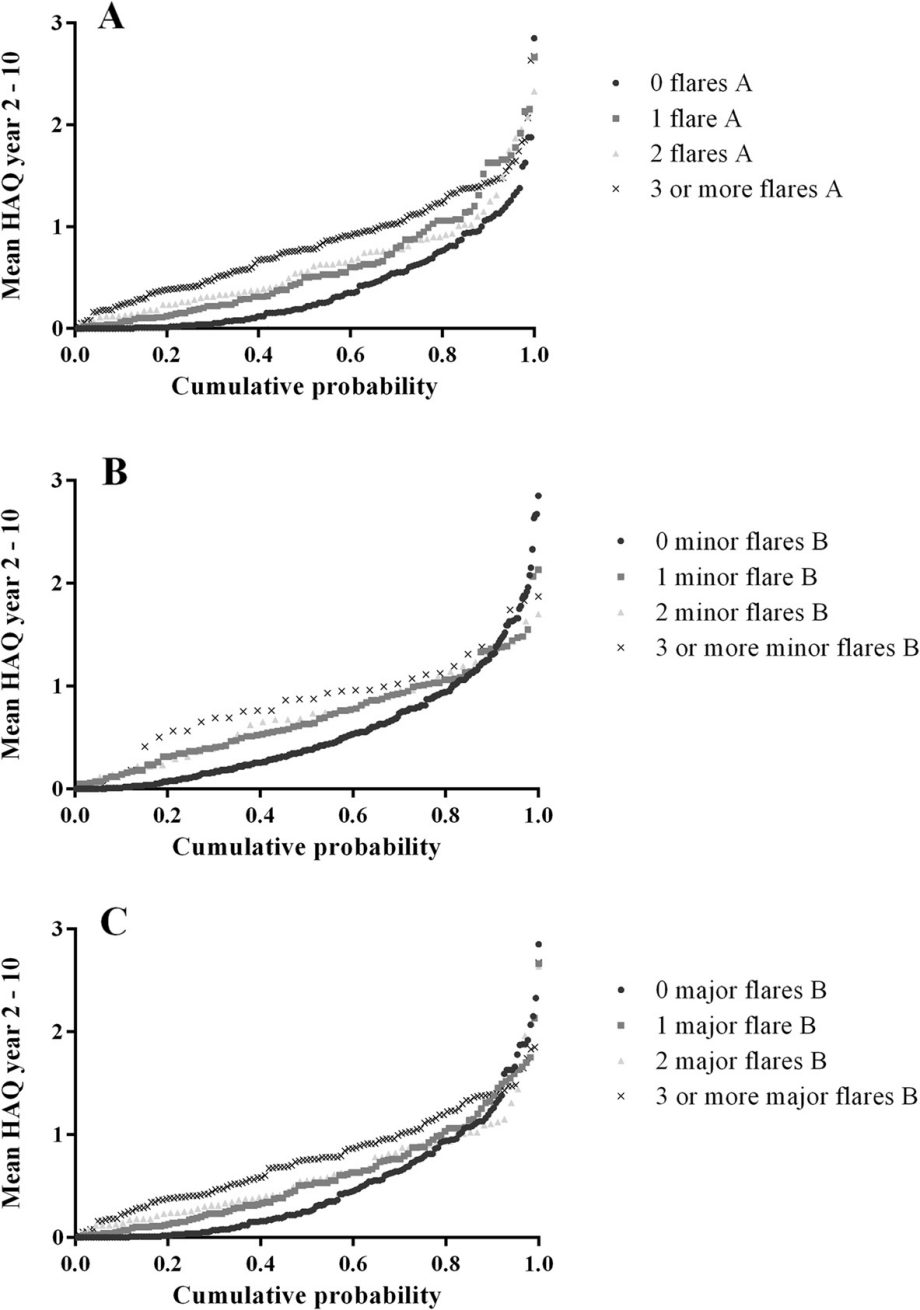
Cumulative probability plots of the mean functional ability (measured with the health assessment questionnaire (HAQ)) during year 2 to year 10 of follow-up, stratified for definition and number of flares. **a** According to the definition of flare A (DAS >2.4, with an increase in DAS of at least 0.6 from a previous DAS of any value). **b** According to the definition of minor flare B (DAS >2.4, from a previous DAS ≤2.4 with an increase of DAS <0.6). **c** According to the definition of major flare B (DAS >2.4 from a previous DAS ≤2.4 with an increase in DAS ≥0.6)

The more flares a patient experienced over time, the higher the mean HAQ over time and the HAQ at year 10 (Table 1 and Fig. 3).

### Visual analogue scales

Increases in each type of VAS were higher in patients with a flare than in patients without a flare, regardless of definition (Table 2). The odds ratios for an increase in VAS ≥20 mm during a flare are reported in Table 2.

**Table 2:** Changes in patient’s assessments of disease activity, pain and morning stiffness and odds ratios for an increase of at least 20 mm in visual analogue scales for these outcomes during the presence of a flare, compared to the absence of a flare (reference category)

	Change in VASda		Change in VASpain		Change in VASms	
	Median	IQR	Median	IQR	Median	IQR
No flare A	0	−8 to 5	0	−8 to 5	0	−8 to 6
Flare A	17	3 to 40	17	3 to 35	11	0 to 29
No flare B	0	−8 to 5	0	−8 to 5	0	8 to 6
Minor flare B	7	−4 to 20	5	−4 to 18	5	−5 to 16
Major flare B	18	4 to 41	18	3 to 37	12	0 to 31
	Increase in VASda^a^		Increase in VASpain^a^		Increase in VASms^a^	
	OR^b^	95 % CI	OR^b^	95 % CI	OR^b^	95 % CI
No flare A	ref	ref	ref	ref	ref	Ref
Flare A	8.47	7.30–9.83	8.35	7.20–9.69	5.63	4.84–6.55
No flare B	ref	ref	ref	ref	ref	Ref
Minor flare B	3.10	2.35–4.07	2.84	2.15–3.75	2.32	1.73–3.12
Major flare B	8.76	7.46–10.28	8.59	7.32–10.08	5.90	5.01–6.94

^a^Increase ≥20 mm from the previous VAS

^b^Adjusted for time

*CI* confidence interval, *IQR* interquartile range, *OR* odds ratio, *ref* reference category, *VASda* visual analogue scale of disease activity, *VASpain* visual analogue scale of pain, *VASms* visual analogue scale of morning stiffness

### Joint damage progression

**Fig. 4:**
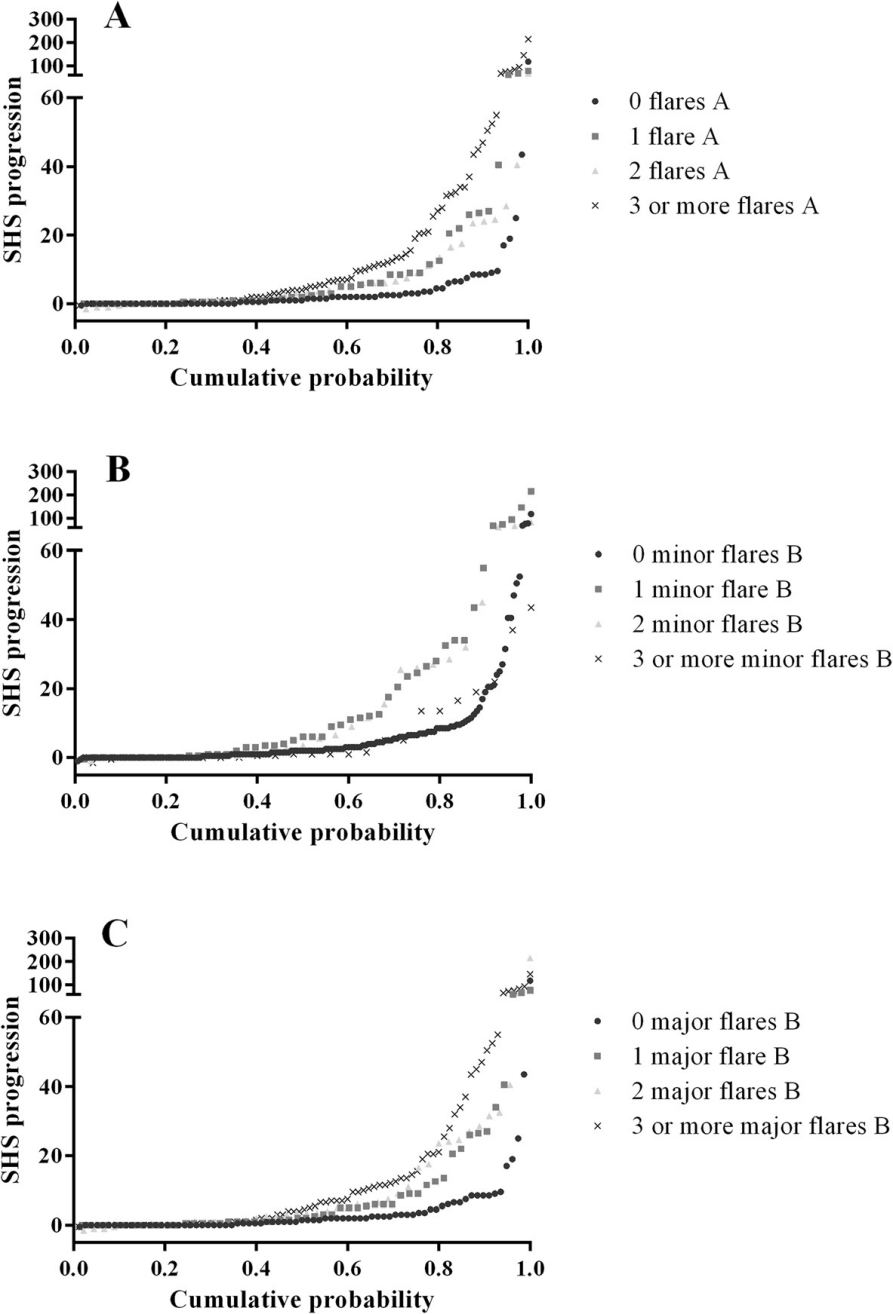
Cumulative probability plots of radiographic progression (measured with the Sharp/ van der Heijde score (SHS)) during 10-year follow-up, stratified for definition and number of flares. **a** According to the definition of flare A (DAS >2.4, with an increase in DAS of at least 0.6 from a previous DAS of any value). **b** According to the definition of minor flare B (DAS >2.4, from a previous DAS ≤2.4 with an increase of DAS <0.6). **c** According to the definition of major flare B (DAS >2.4 from a previous DAS ≤2.4 with an increase in DAS ≥0.6)

Over 10 years, SHS progression in completers was lowest in patients without any flare during follow-up, and increased with the number of flares A and major flares B (Table 1 and Fig. 4). Proportions of patients without any radiographic progression can be derived from Fig. 4.

Over time, during a year where a flare A occurred, the adjusted odds ratio (OR) of developing SHS progression was 1.74 (95 % confidence interval (CI) 1.07–2.85; *p* = 0.027), compared to no flare A as reference category. Patients with a minor flare B had an adjusted OR of 2.11 (95 % CI 0.87–5.13; *p* = 0.101) to develop SHS progression and patients with a major flare B had an adjusted OR of 1.72 (95 % CI 1.01–2.91; *p* = 0.044), both compared to the absence of a flare B.

## Discussion

In this post-hoc analysis of the BeSt study, we determined the short-term and long-term effects of flares defined using the original DAS. During a flare, functional ability decreased and patients reported higher VAS for disease activity, pain and morning stiffness. In addition, joint damage progression occurred more often when a patient experienced a flare during that year. Long-term assessments showed a higher loss of functional ability and more radiographic progression in patients who had suffered a flare, and there was a dose–response relation with the number of flares over time.

The definition of a flare in RA is still in development [1–3]. There may be transient periods of symptoms for which patients do not contact their rheumatologist. It is, however, determined that flares involve a deterioration of patient-reported outcomes, such as functional ability and VAS of general health, pain and morning stiffness [4, 6]. It has also been suggested that DAS28 is a useful instrument to define flares [5], and that registration of a flare should be followed by treatment intensification [3–6]. However, treatment intensification for a flare that may spontaneously resolve might constitute overtreatment. Therefore we monitored short- and long-term disease outcomes in relation to the occurrence of flares in the BeSt study.

We formulated three definitions of a disease flare based on the original DAS as obtained in the BeSt study. Over 10 years, besides the DAS measurements, functional ability was assessed 3-monthly using the HAQ, and patient’s assessments of pain, disease activity and morning stiffness on a VAS were registered. Comparable prevalence of flare A and major flare B were found, as a result of overlapping definitions (Fig. 2). Minor flare B occurred less often. This might be explained by the rather strict definition, which required an increase of DAS to above 2.4 but of less than 0.6 compared to the previous DAS. Still, there was a statistically significant increase in HAQ in case of a minor flare B, although this was not a clinically relevant increase, and a trend was seen for increasing number of flares with decreasing functional ability (Table 1 and Fig. 3). In 11 % of minor flare B, rheumatologists did not adjust medication. In 73 % of these cases the next DAS was again ≤2.4. This appears to illustrate that (minor) flares will spontaneously remit and need no additional therapy. We have to stress, however, that these are very small numbers and, if true, then only for minor flares. Numerically, there appeared to be slightly more joint damage progression in patients who suffered a minor flare B compared to patients who never experienced a minor flare B (Fig. 3), although a dose–response relationship could not be demonstrated. The dose–response relationships should be interpreted carefully anyhow, as individual patients may have various types of flares in various frequencies.

The treatment protocol required stepwise tapering and discontinuation of antirheumatic drugs if the DAS was ≤2.4 during at least 6 months. We wondered if reducing medication could have triggered flares. Only in 25 % of the flares, by whichever definition, had medication been tapered at the preceding visit. This could be linked to our finding of a higher flare prevalence during the early years of follow-up and decreasing prevalence in the later years (Fig. 1). It was previously reported that during year 1 and 2 of follow-up, when a low DAS was achieved, and particularly if DAS was ≤2.4 twice in a row, there was a high probability that the next DAS would also be ≤2.4 [19]. During later years of follow-up, this probability was even higher, up to 95 % (data not shown). The decrease shown in Fig. 1 can be either an overestimation or an underestimation, as patients with sustained (drug-free) remission are more likely to drop out [20] and, although patients were instructed to visit their rheumatologist at the moment of a flare, flares between two study visits might have been missed. Despite this, we suggest that when a treat-to-target strategy is adopted from the start and continued over time, RA may become relatively indolent in most patients. Since a dose–response relationship between the number of flares and the degree of long-term functional disability and joint damage was demonstrated, one could also hypothesize that targeted treatment should be even stricter than required in the BeSt study. Rheumatologists need to be further encouraged to adjust medication each time a flare is registered rather than hope for a spontaneous improvement. In addition, on the condition that protocol violations would not occur even more often, the treatment target may be set lower, for instance at remission [10]. The disease activity over time may then be lower and episodes of high disease activity more rare. However, this comes with a risk of overtreatment and, potentially, with higher turnover of medication in some patients, and considerable costs. Setting the treatment target lower will also influence the definition of flare.

A recent study examined the frequency of flares in a cohort of patients with established RA [21]. Flares were self-reported by the patients through 6-monthly questionnaires. During 3 years of follow-up, 99 % of the patients reported at least one flare, with a frequency of 54–74 % per evaluation. These percentages suggest a higher flare frequency than in our study (67–69 % of our patients had at least one flare during 9 years; frequency 4–11 % per visit). Possibly, the notion of a flare as experienced by patients only partially overlaps with our DAS-based flare definitions. Also, the reported flares were inventoried retrospectively, whereas we relied on DAS increases as measured at the time of the study visits. Since Bykerk et al. reported that patients with higher disease activity tended to report more flares [21], the fact that the majority of our patients had low disease activity during most of the observation time may have conditioned a reduced flare frequency [22].

We may have underestimated the prevalence of flares, as we missed short-term subjective flares by focusing on 3-monthly DAS measurements for our flare definition. This will, however, not affect the associations found between the presence of flares and functional ability loss, increase in VAS scores, and joint damage progression. Another limitation of our study could be the amount of missing data. Patients have dropped out of the study (up to 38 % at year 10) or may have missed some visits. To avoid the influence of missing data, we performed mixed models. This approach takes into account the correlation of repeated measurements within a patient and between variables when handling the missing data.

## Conclusion

In patients with RA a flare in disease activity is associated with functional disability, more pain and morning stiffness, and more radiographic progression, both in the short-term and the long-term. Therefore, it seems appropriate to intensify therapy after each flare. Any risk of overtreatment in case of a disease flare that would spontaneously remit may be less serious than the risk of undertreatment resulting in long-term disability and joint damage. Continued targeted therapy might reduce the frequency of flares, suggesting that with an adequate treatment strategy RA may become more indolent. In that case, it is possible that tight monitoring in patients who achieved persistent low disease activity may be exchanged for longer monitoring intervals, under the condition that patients who suspect a flare can be readily assessed and treatment can be adjusted if necessary.

## Abbreviations

CI: Confidence interval
DAS: Disease activity score
DAS28: 28-joint disease activity score
EULAR: European League Against Rheumatism
GLMM: Generalized linear mixed model
HAQ: Health assessment questionnaire
IQR: interquartile range
LMM: Linear mixed model
OR: Odds ratio
RA: Rheumatoid arthritis
SD: standard deviation
SHS: Sharp/ van der Heijde score
VAS: Visual analogue scale
VASda: visual analogue scale of disease activity
VASgh: visual analogue scale of general health
VASms: visual analogue scale of morning stiffness
VASpain: visual analogue scale of pain
